# An Investigation of Deep Learning Models for EEG-Based Emotion Recognition

**DOI:** 10.3389/fnins.2020.622759

**Published:** 2020-12-23

**Authors:** Yaqing Zhang, Jinling Chen, Jen Hong Tan, Yuxuan Chen, Yunyi Chen, Dihan Li, Lei Yang, Jian Su, Xin Huang, Wenliang Che

**Affiliations:** ^1^Department of Cardiology, Shanghai Tenth People's Hospital, Tongji University School of Medicine, Shanghai, China; ^2^Department of Software Engineering,School of Informatics Xiamen University (National Demonstative Software School), Xiamen, China; ^3^Department of Computer and Software, Institute of System Science, National University of Singapore, Singapore, Singapore; ^4^Nanjing University of Information Science and Technology, Nanjing, China; ^5^School of Software, Jiangxi Normal University, Nanchang, China

**Keywords:** EEG, emotion recognition, DNN (deep neural network), CNN (convolutional neural network), CNN-LSTM

## Abstract

Emotion is the human brain reacting to objective things. In real life, human emotions are complex and changeable, so research into emotion recognition is of great significance in real life applications. Recently, many deep learning and machine learning methods have been widely applied in emotion recognition based on EEG signals. However, the traditional machine learning method has a major disadvantage in that the feature extraction process is usually cumbersome, which relies heavily on human experts. Then, end-to-end deep learning methods emerged as an effective method to address this disadvantage with the help of raw signal features and time-frequency spectrums. Here, we investigated the application of several deep learning models to the research field of EEG-based emotion recognition, including deep neural networks (DNN), convolutional neural networks (CNN), long short-term memory (LSTM), and a hybrid model of CNN and LSTM (CNN-LSTM). The experiments were carried on the well-known DEAP dataset. Experimental results show that the CNN and CNN-LSTM models had high classification performance in EEG-based emotion recognition, and their accurate extraction rate of RAW data reached 90.12 and 94.17%, respectively. The performance of the DNN model was not as accurate as other models, but the training speed was fast. The LSTM model was not as stable as the CNN and CNN-LSTM models. Moreover, with the same number of parameters, the training speed of the LSTM was much slower and it was difficult to achieve convergence. Additional parameter comparison experiments with other models, including epoch, learning rate, and dropout probability, were also conducted in the paper. Comparison results prove that the DNN model converged to optimal with fewer epochs and a higher learning rate. In contrast, the CNN model needed more epochs to learn. As for dropout probability, reducing the parameters by ~50% each time was appropriate.

## Introduction

There are many research methods applied to real-time emotion recognition. For example, researchers use electroencephalogram (EEG) signals and peripheral physiological such as ECG, respiration, skin resistance, and blood pressure to carry out emotion recognition research (Horlings et al., 2008). Among them, the EEG signal in the objective physiological signal is directly generated by the central nervous system, which is closely related to human emotional states (Jiang et al., 2020b).

There are usually two machine learning strategies for analyzing EEG signals: step-by-step machine learning and end-to-end deep learning (Yang et al., 2020). Step-by-step machine learning mainly involves three steps: the first step is to obtain the digital data by sampling the analog signals, known as signal preprocessing. The second step is artificial feature extraction, which is to calculate the features using feature extraction formulas. Finally, the features are classified using mechine learning methods to achieve the emotion classification result. Wavelet transform and entropy measures are widely used in feature extraction (Zhang et al., 2018). Murugappan et al. (2010) used the “db4” wavelet function for deriving a set of conventional and modified energy-based features from EEG signals for classifying emotions. Paul et al. (2015) used the multifractral detrended fluctuation analysis (MFDFA) method to extract features and used a support vector machine (SVM) to categorize the EEG feature space related to various emotional states into their respective classes. Jiang et al. (2020a) used transfer learning to reduce the differences in data distribution between the training and testing data (Yang et al., 2016; Jiang et al., 2017). Besides, they proposed a novel negative-transfer-resistant fuzzy clustering model (Jiang et al., 2015) with a shared cross-domain transfer latent space (Jiang et al., 2019).

However, it is difficult to cover all the implied features by manual extraction, and the formulae used to extract time-domain and frequency-domain features are often very complex. In addition, EEG signals are susceptible to noises such as electromyographic artifacts, which create serious interference in the progressive machine learning approach. In view of the above situations, some end-to-end deep learning methods are used to solve these problems. Alhagry et al. (2017) proposed a long short-term memory (LSTM) model to learn features from EEG signals. The classification accuracy reached 85.65, 85.45, and 87.99% for different labels. Schirrmeister et al. (2017) used convolutional neural networks (CNN) for EEG decoding and visualization which have shown great potential when applied to end-to-end emotion recognition based on EEG-signals. Zhang et al. (2017) improved the entirely automatic feature extraction of MWL classification which then had effective high performance compared with traditional machine learning methods.

Since the deep learning models for EEG-based emotion recognition are still in their infancy, there is still a lot of room for adjustment in model structure and parameter settings. In this paper, we investigated the application of existing deep learning models widely used in this field, and implemented several popular deep learning models including deep neural networks (DNN), convolutional neural networks (CNN), long short-term memory (LSTM), and a hybrid model of convolutional neural networks and long short-term memory (CNN-LSTM) for EEG emotion recognition.

Firstly, we extracted 63 s of 32-channel EEG data from 40 trials of 32 subjects. In order to improve the classification efficiency, we selected 14 channels which were most suitable for EEG emotion classification. We built two feature datasets, including RAW data and standard (STD) data. STD data were extracted by calculating eight eigenvalues including the maximum, standard deviation, kurtosis, and so on. In order to make efficient use of the data, we used a 10-fold cross-validation method to build the dataset for each sample before training the model. We put the pre-processed feature datasets into four deep learning models, classifying the emotion in the valence dimension and arousal dimension to four labels by one-hot encoding. In terms of model design selection, we adjusted the learning rate, epoch, and dropout probability, and compared the applicable values of different models. Finally, we obtained the comparison results of the four models.

The rest of this article is organized as follows:

Data description, data preprocessing, deep learning models for emotion recognition, experiments, conclusion, and references.

## Data Description

The experiment was carried out on the DEAP dataset (Koelstra et al., 2012). The dataset was developed by a team of researchers at Queen Mary University of London and is a large multimodal database for the analysis of spontaneous emotions. It contains EEG, ECG, EMG, and other peripheral physiological signals. To collect the signals, 32 subjects were asked to watch 40 segments of 1-min music videos which represented different emotions. Their corresponding brain signals were collected as they watched the videos. After watching each video, participants rated their emotional responses to the 40 music videos on a scale of one to nine based on excitement, control, and how much they liked and were familiar with the videos.

The DEAP database includes two parts: online evaluation and physiological experiment. The online evaluation mainly contains basic information about the initial stimulus material. The physiological experiment mainly contains information on the experiment, including the number of experimental subjects, the rating values, and the recorded signals. The rating scales include arousal, valence, dominance, liking, and familiarity. The rating value from small to large indicates that each index is from negative to positive, from weak to strong.

The DEAP dataset contains 32 groups of EEG data in total, corresponding to the experimental data of 32 subjects (s01–s32). The data of each subject contains two arrays: ***data*** and ***labels***.

## Data Preprocessing

### The Pre-processing of RAW Data

In this paper, the ***data*** array we used had already been preprocessed, in which the EEG data were desampled, the sampling frequency became 128 Hz, and then the signal was filtered to 4–45 Hz through a band-pass filter. Then the EEG data were averaged to the same reference. We chose 63 s as the reference length of each trail, of which the first 3 s were the preparing time, and the other 60 s were collected during the watching of the video. Therefore, in every separate trail, there were 63 s^*^128 Hz = 8,064 sampling points for each channel.

**Figure 1:**
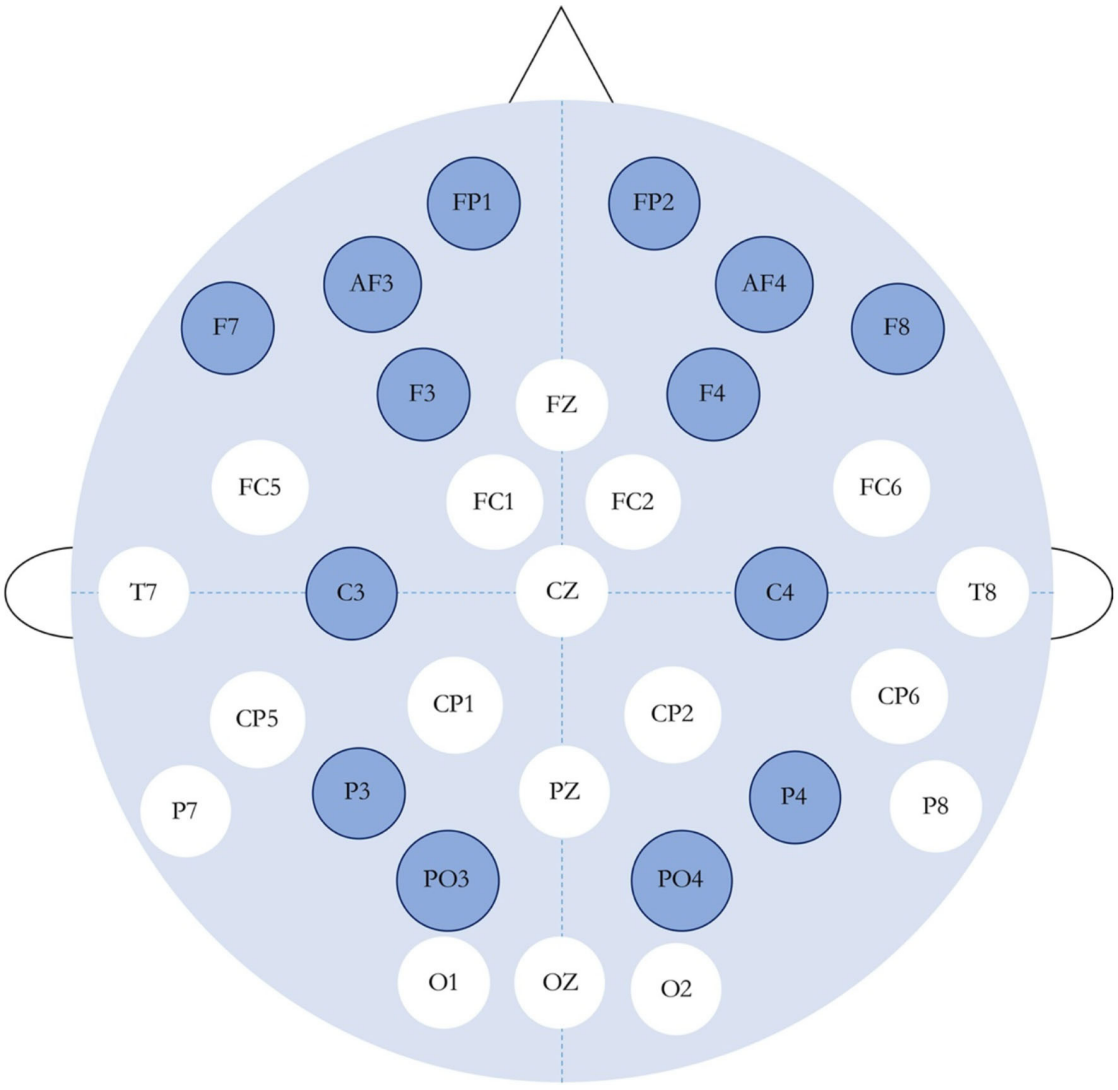
International 10–20 standard system electrode position distribution map (the dark-colored ones are the channels used in this experiment).

In order to simplify the training difficulty and improve the accuracy of emotion recognition, we selected 14 corresponding electrodes of the channels which had the most significant impact on the generation of emotion. Table 1 shows the 14 selected channels and their corresponding electrodes. Figure 1 shows the international 10–20 standard system electrode position distribution map, in which the 14 selected electrodes were labeled with different colors to indicate the different influence on emotion generation.

**Table 1:** The selected EEG channels.

**Channel no**.	**Channel content**
1	Fp1
2	AF3
3	F3
4	F7
7	C3
11	P3
13	PO3
17	Fp2
18	AF4
20	F4
21	F8
25	C4
29	P4
31	PO4

The total EEG cycles were 40 tests per subject. We set the data dimension of the dataset to 40 (epochs)^*^14 (channels)^*^8,064 (time points). In order to distinguish from the feature extraction data indicated later, we used RAW data to represent this 40 × 14 × 8,064 data array.

### The Pre-processing of STD Data

Feature extraction is necessary in the construction of a deep learning neural network for an emotion recognition task. Of all the domains, we chose the characteristics of time and frequency as the data features. Taking the time point of the raw data array processed in the previous section as the raw time domain feature forms **RAW** data.

We used eight statistical methods to extract the features of the time domain. The following mathematical formula shows how these features were calculated, where E(n) stands for the signal value of the *n* time points.

The mean of the original signal:

(1) $$\begin{array}{l} \mu_{E}=\frac{1}{N}\overset{N}{\underset{n=1}{\sum }}E\left(n\right) \end{array}$$

The standard deviation of the original signal:

(2) $$\begin{array}{l} \sigma_{E}=\sqrt{\frac{1}{N}\overset{N}{\underset{n=1}{\sum }}{\left(E\left(n\right)-\mu_{E}\right)}^{2}} \end{array}$$

The maximum of the original signal:

(3) $$\begin{array}{l} E_{max}=\max E\left(n\right) \end{array}$$

The minimum of the original signal:

(4) $$\begin{array}{l} E_{min}=\min E\left(n\right) \end{array}$$

Average absolute value of the first difference:

(5) $$\begin{array}{l} \delta_{E}=\frac{1}{N-1}\overset{N-1}{\underset{n=1}{\sum }}|E\left(n+1\right)-E\left(n\right)| \end{array}$$

Average absolute value of the second difference:

(6) $$\begin{array}{l} \lambda_{E}=\frac{1}{N-2}\overset{N-2}{\underset{n=1}{\sum }}|E\left(n+2\right)-2E\left(n+1\right)+E\left(n\right)| \end{array}$$

The skewness of the original signal (Mardia, 1970):

(7) $$\begin{array}{l} Skew=\frac{1}{N}\overset{N}{\underset{n=1}{\sum }}\left[{\left(\frac{E\left(n\right)-\mu_{E}}{\sigma_{E}}\right)}^{3}\right] \end{array}$$

The kurtosis of the original signal (deCarlo, 1997):

(8) $$\begin{array}{l} Kurtosis=\frac{N\left(N+1\right)}{\left(N-1\right)\left(N-2\right)\left(N-3\right)}\overset{N}{\underset{n=1}{\sum }}{\left(\frac{E\left(n\right)-\mu_{E}}{\sigma_{E}}\right)}^{4}\\ \;-\frac{3{\left(N-1\right)}^{2}}{\left(N-2\right)\left(N-3\right)} \end{array}$$

The combination of the eight statistical features into an eigenvector to represent the time domain features are known as the **STD** data. We divided the 63 s of each trail into 1 s segments, each segment contained 128 sampling points. Then the **STD** data array shape was 40(epoch) ^*^ 14 (channel) ^*^ 63 (time segment) ^*^ 128 (sampling point).

To compare the performance of different feature extraction methods under different deep learning neural networks, we used **RAW** and **STD** as two different features to train the models.

### The Description of Data and Label

The labels array was a 40 × 4 two-dimensional array, which represented the video/trial × label (valence, arousal, dominance, liking) corresponding to the self-evaluation of each MV. The **valence** level (on a scale of 1 to 9) indicated how happy people felt. People with a happier mood were tested in a higher valence. The **arousal** level (on a scale of 1 to 9) represented the activation of feeling in people. People with a high level of activation generated a higher arousal rating. In this paper, we considered **valence** and **arousal** as the two dimensions of measurement to classify the emotions of the subjects.

**Figure 2:**
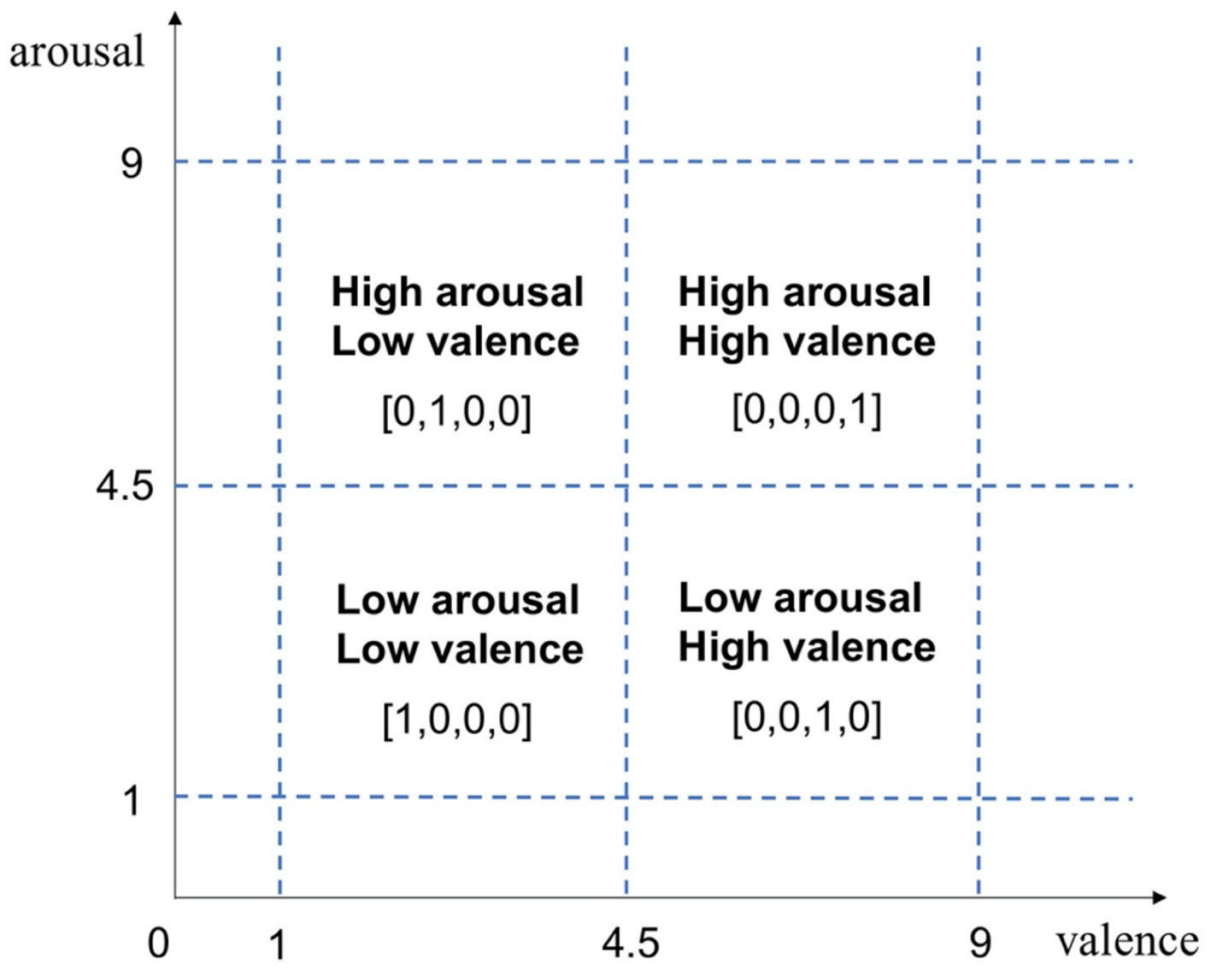
Valence/arousal measurement and one-hot encoding labels.

In order to transform the continuous rating into a discrete tag form, we used the one-hot encoding form to classify the four types of emotions, as shown in Figure 2. The emotions of the subjects were divided into four categories: high arousal/high valence, high arousal/low valence, low arousal/high valence, and low arousal/low valence, which were expressed as [0,0,0,1], [0,1,0,0], [0,0,1,0], and [1,0,0,0]. Thus, the shape of the ***Labels*** array was 40 (epochs) × 4 (label category).

This paper uses ***RAW data***, ***STD data*,** and ***Labels*** as inputs for the neural network. The array description is shown in Table 2.

**Table 2:** Data description.

**Array name**	**Array shape**	**Array contents**
RAW data	40 × 14 × 8,064	trial no. × channel data
STD data	40 × 14 × 63 × 128	trial no. × time segment × sampling point
Label	40 × 4	trial no. × label (one-hot encoding)

## Deep Learning Models for Emotion Recognition

In this paper, we investigated widely used deep learning models for emotion recognition based on EEG signals. We implemented several popular deep learning models including a DNN, CNN, and LSTM for RAW data classification, and designed CNN-LSTM models for higher performance.

### Deep Neural Network Model

In the traditional sense, the neural network is also called a multi-layer perceptron. It is composed of an input layer, an output layer, and a number of hidden layers. Continuous functions such as Sigmoid or Tanh are used to simulate the response of neurons to excitation. The multi-layer perceptron can remove the constraint of the earlier discrete transmission function. However, as the number of layers increases, the optimization function is more and more likely to fall into the local optimal solution. On the basis of the multi-layer perceptron, the DNN replaces the *Sigmoid* function with *ReLU, maxout*, and other activation functions, effectively overcoming the gradient disappearance problem (Hanin, 2019).

In this paper, we mainly used a fully connected **DNN** model as the basic model in emotion classification.

### Convolutional Neural Network Model

A convolutional neural network (CNN) has been applied widely in original signal processing and image recognition. A CNN has three significant characteristics: a local sensing field, weight sharing, and down sampling, which can decrease the complexity of the network. The high accuracy of the recognition tasks is mainly because it can learn local non-linear features by convolution and non-linear activation functions, and express high-level features as the combination of low-level features. In addition, many CNNs use a pooling layer to create a rough representation of intermediate features, which makes the CNN more translation invariant (Chen et al., 2019).

**Figure 3:**
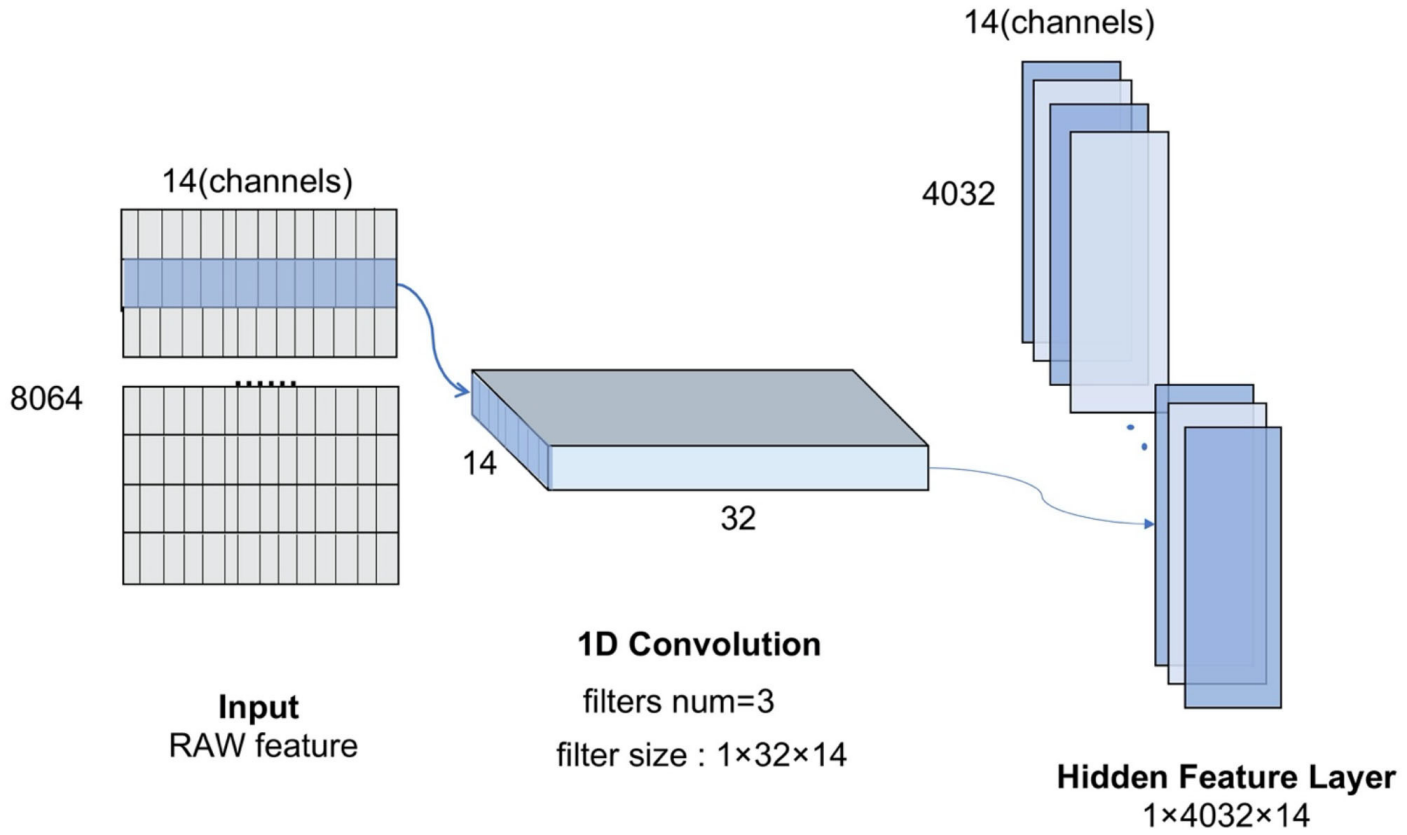
The convolution-max process diagram.

For the CNN model, the convolution kernel is the key to automatic feature extraction (Cheng and Parhi, 2020). Figure 3 shows the 1D-convolution kernel for the automatic feature extraction of our model.

The ReLU function is usually used as the activation function due to its simplicity of implementation. It can speed up calculation and convergence. The ReLU function has the following formula:

(9) $$\begin{array}{l} f\;\left(x\right)=\;\max\left(0,\;x\right) \end{array}$$

**Figure 4:**
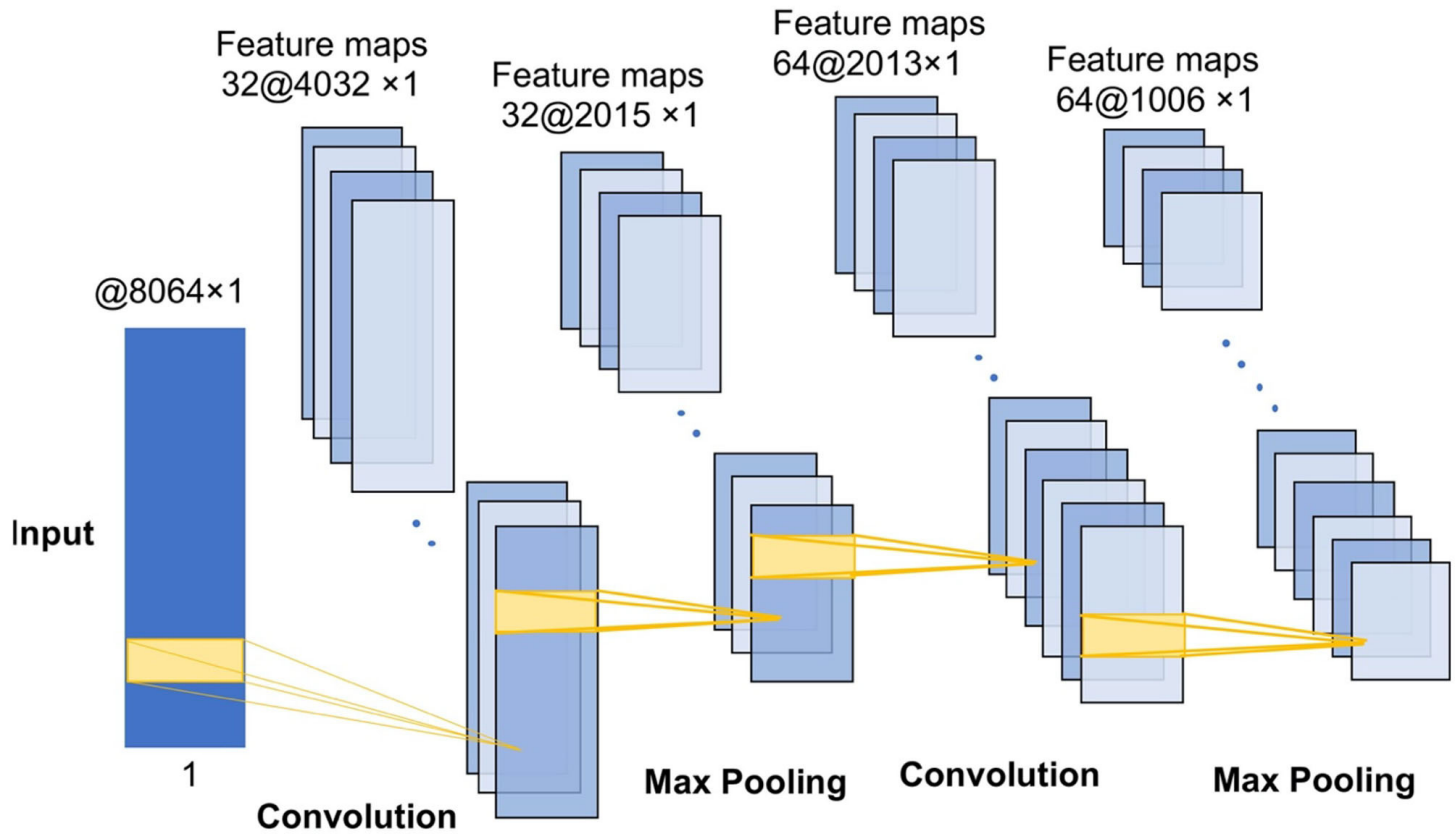
The convolution-max process diagram.

The pooling layer is a structure for down sampling the features obtained from the convolutional layers, which can reduce the amount of computation and the degree of over-fitting of the network to some extent, thus improving the peKrformance of the CNN model. The convolution-max-pooling process is shown in Figure 4.

**Figure 5:**
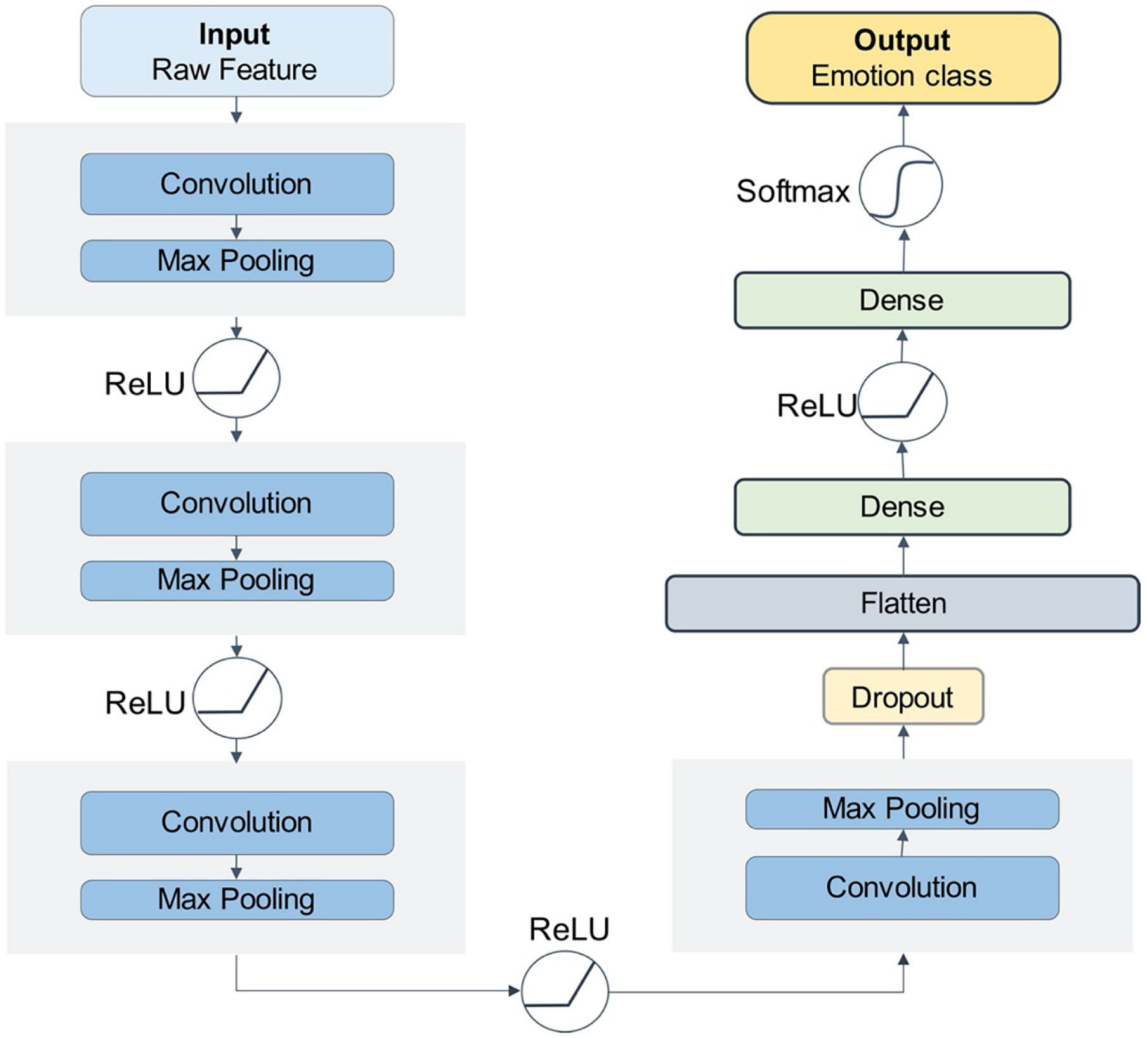
The CNN model structure.

Our CNN model has four convolution-max-pooling blocks, then a Flatten layer to expand the convolution results, followed by two full-connection layers, and a dense softmax layer. This configuration is the optimal model for the DEAP emotion classification dataset. Figure 5 shows the structure of our deep CNN model.

### Long Short-Term Memory Model

A recurrent neural network (RNN) is a type of recursive neural network that inputs sequential data and performs recursion in the evolutionary direction of a sequence, connected by a chain of all the recurrent units (Graves et al., 2013). An LSTM network is a variant of the recurrent neural network, which is mainly used to process sequence information with a long time difference.

**Figure 6:**
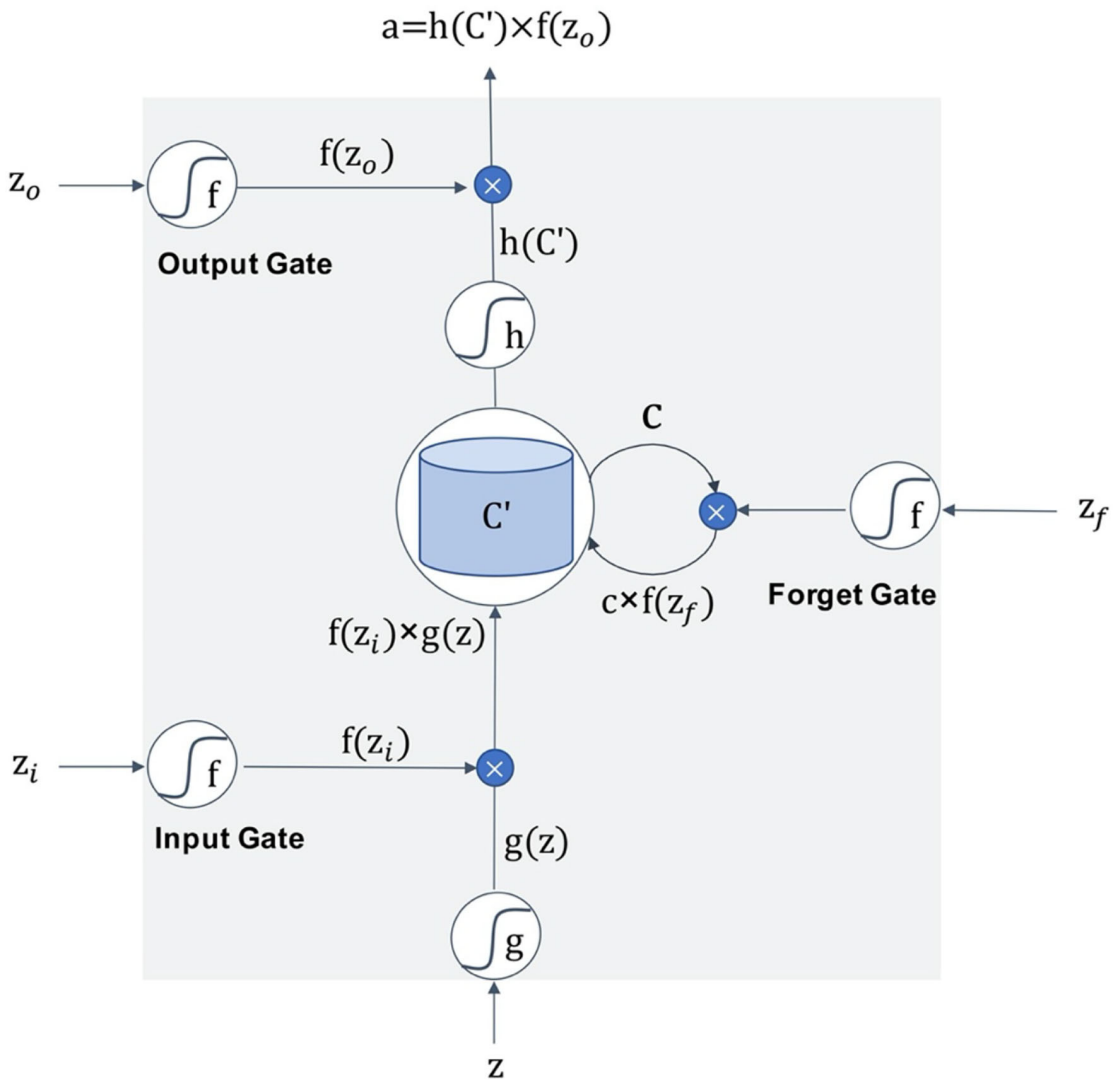
The schematic diagram of the LSTM unit.

**Figure 7:**
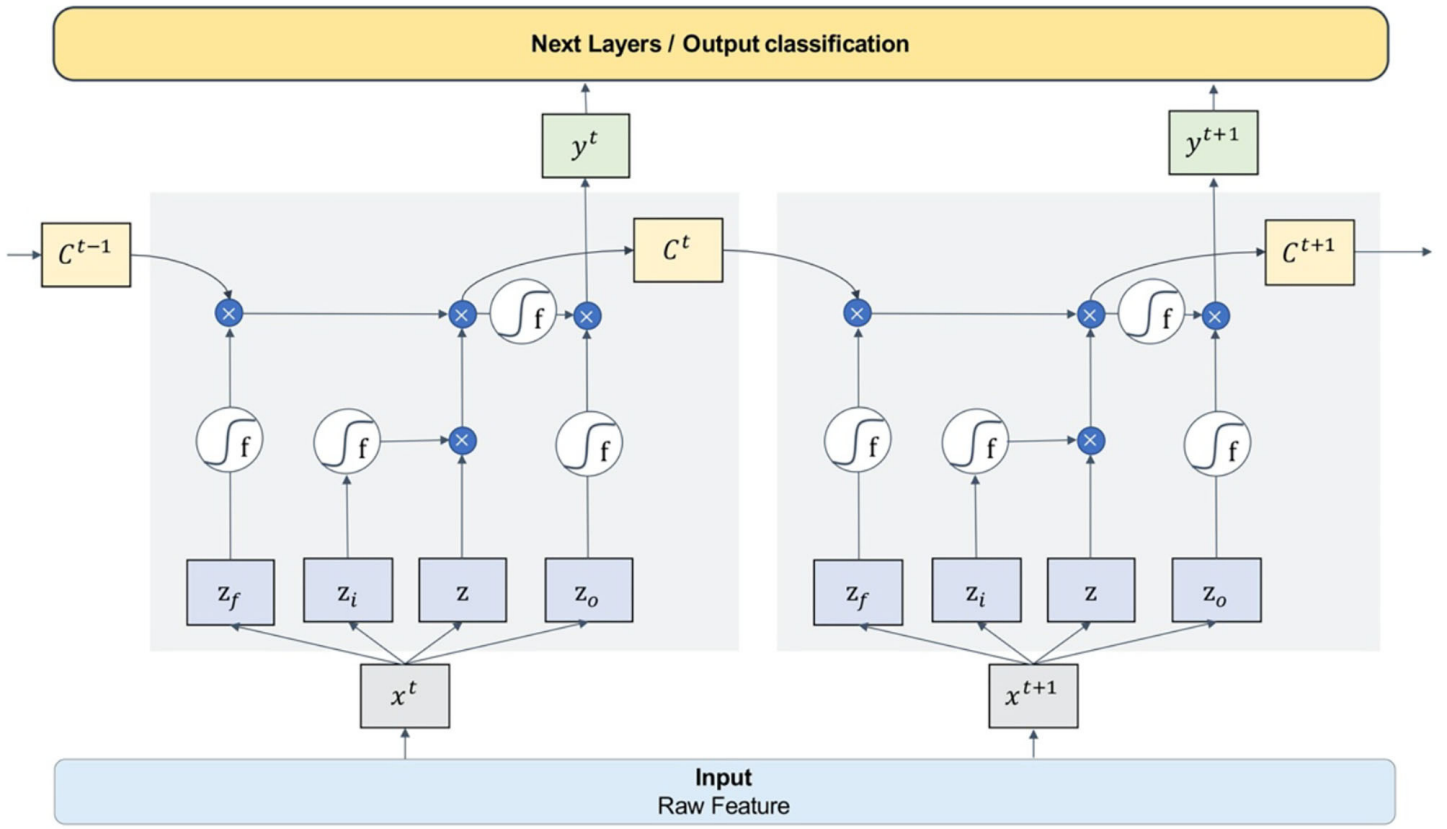
The connection mode and working principle of the LSTM model.

Figure 6 is the schematic diagram of the LSTM unit. The LSTM unit inputs four variables from one input entry and three other gates, which is different from the neuron univariate input of other models. For each neuron in the neural network, the door is opened or closed by the value of input data and parameter weight, and these parameters can be obtained by model training. An LSTM network can solve the problem of gradient vanishing in back propagation by adding three gates. Many of these units are linked together in time series and can form an LSTM model, as the Figure 7 shows.

In this paper, we designed a four-layer LSTM network structure. The model takes **RAW** data as input, passes through four layers of the one-way LSTM network, then connects to a dropout layer, and finally reaches the full connection classification layer.

### CNN-LSTM Model

The CNN is good at extracting the spatial local relevant features of data, but it struggles to capture the long-term dependence relationship in sequence data, which can be remedied by the LSTM. So, a hybrid model of the CNN and LSTM have been proven to have good performance in natural signal recognition (Ma and Hovy, 2016; Zhao et al., 2019). Therefore, this paper proposed a CNN-LSTM hybrid network model of the CNN and LSTM serial.

**Figure 8:**
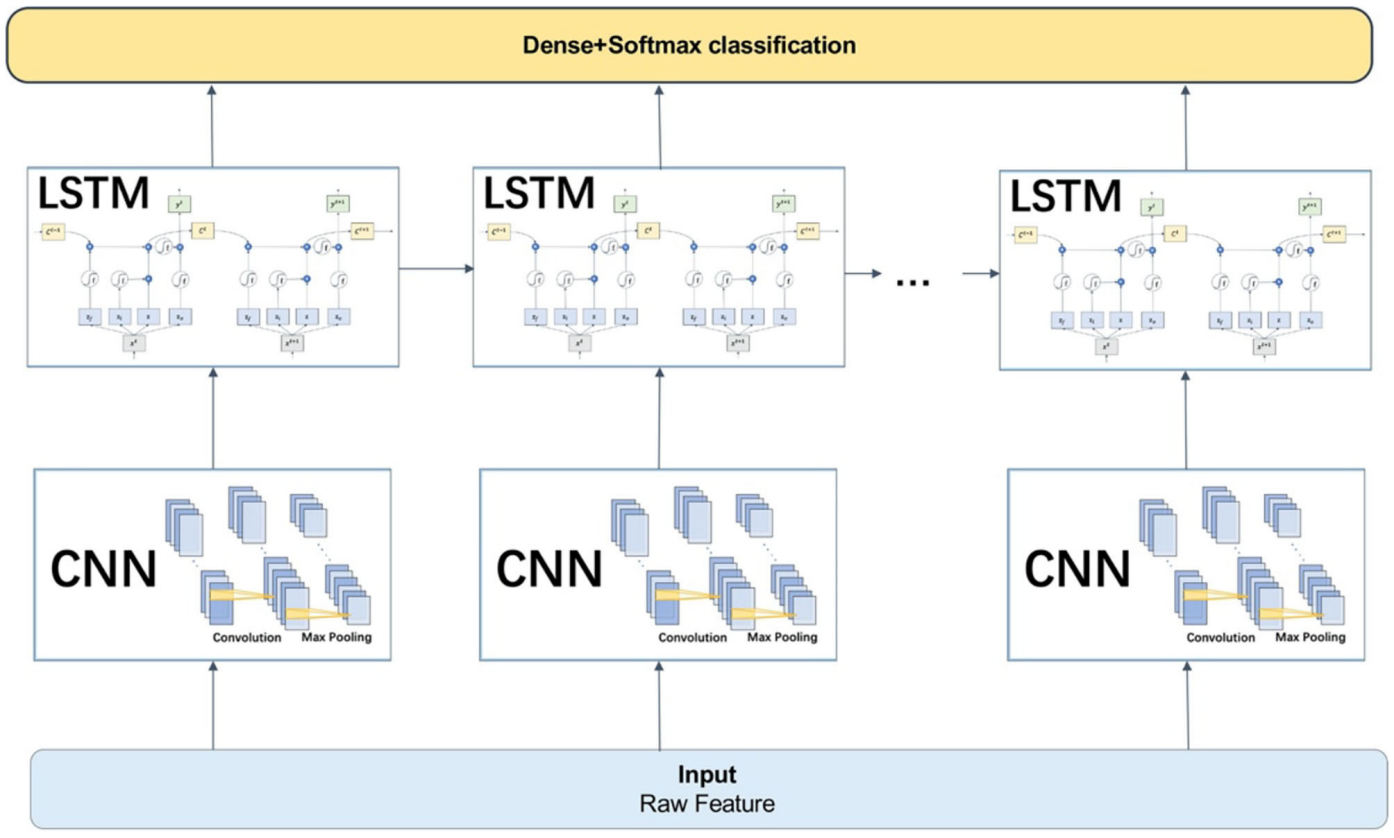
The connection mode and working principle of the CNN-LSTM model.

In the **CNN-LSTM model**, the **RAW data** were taken as input and used the CNN model for feature extraction before entering the LSTM. After taking the input from the CNN layer, the LSTM units connected as a link and passed the result to the next layer, usually the fully connected dense layer and the softmax classification layer, see Figure 8.

The LSTM of the first layer contained 64 units and the second layer contained 32 units. We chose ReLU as the activation function of the LSTM layer in order to prevent the gradient vanishing. In addition, since the LSTM model had many more parameters than the other deep learning models, we added two dropout layers to prevent over-fitting of the training data. Finally, the full connection layer and the softmax function were used as the classification output layer.

## Experiments

### Experimental Setup

In the experiments, we implemented the DNN, CNN, LSTM, and CNN-LSTM, respectively, adjusted the structural design of the model, and optimized the parameters. In addition, it was mentioned that there are two machine learning strategies for analyzing EEG signals: step-by-step machine learning and end-to-end deep learning. In order to compare the two feature extracting methods, we took the **RAW** data and **STD** features, which had been pre-processed, as the input data of the models, respectively.

In terms of training models, we used the method of ***10-fold cross-validation*** to train the classifier. The original data were divided into 10 subsamples, with one subsample retained as the validation set and the other nine samples used as the training set. The cross-validation was repeated 10 times to create an average of the estimation results. This validation method helped us obtain a more reliable and stable model by using limited label data. We chose ***Adam*** as the optimizer of the models (Shindjalova et al., 2014).

To evaluate the classification performance of the model, we used ***Acc*** and ***Loss*** to represent the accuracy and loss. The calculation formula of ***Acc*** is:

(10) $$\begin{array}{l} Acc=\frac{\text{CP}+\text{CN}}{\text{CP}+\text{CN}+\text{MN}+\text{MP}} \end{array}$$

In this formula, CP is the number of positive examples that were correctly classified, CN is the number of negative examples that were correctly classified, MN is the number of positive examples that were misclassified, and MP is the number of negative examples that were misclassified.

Because the classification options are one-hot encoding multi-dimensional vectors, we used ***multi-classification cross-entropy*** as the loss function for all the models (de Boer et al., 2005). The Loss of the model can be calculated by the following formula (Zhang et al., 2021):

(11) $$\begin{array}{l} Loss=-\overset{T}{\underset{j=1}{\sum }}y_{i}logP_{j} \end{array}$$

where *y*_*j*_ is the classification of the *j* th sample and *P*_*j*_ is the probability of the *j* th sample to be recognized as *y*_*j*__._

### Accuracy and Comparison Results

**Figure 9:**
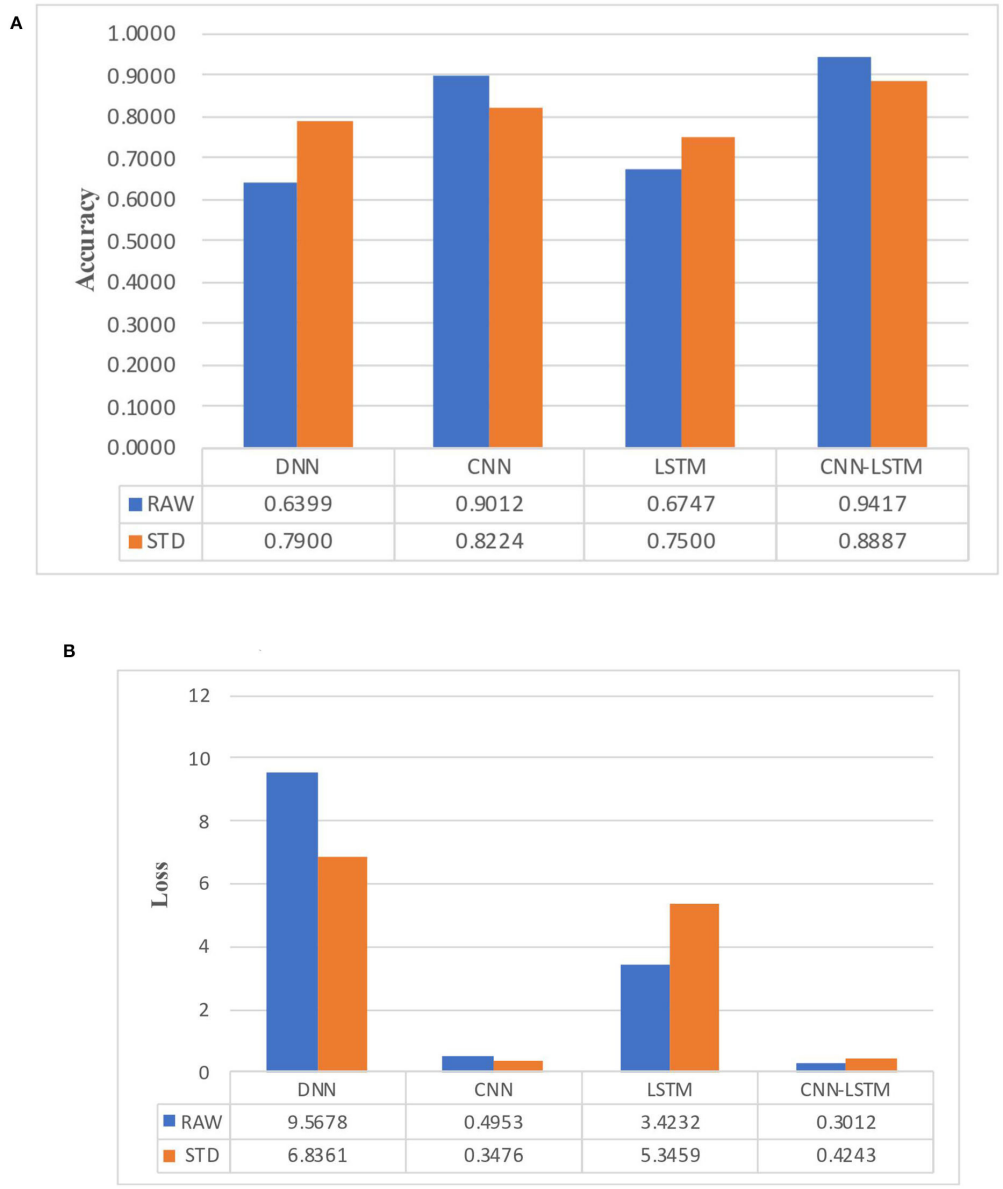
Performance comparison histogram between different models. **(A)** Classfication accuracy of different models. **(B)** Classification loss of different models.

The training and test set included 55% positive samples and 45% negative samples, so it can be considered that the number of positive and negative samples was basically consistent. We repeated each model testing five times and averaged the results of those tests. The Tables 3, 4 show the experimental results of ***Acc*** and ***Loss***. Figure 9 shows a bar chart comparison of them.

**Table 3:** The *Acc* result of different models and different features.

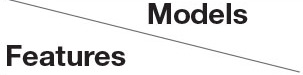	**DNN**	**CNN**	**LSTM**	**CNN-LSTM**
RAW	0.6399	0.9012	0.6747	0.9417
STD	0.7900	0.8224	0.7500	0.8887

**Table 4:** The *Loss* result of different models and different features.

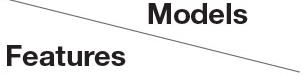	**DNN**	**CNN**	**LSTM**	**CNN-LSTM**
RAW	9.5678	0.4953	3.4232	0.3012
STD	6.8361	0.3476	5.3459	0.4243

### Additional Analysis and Results

During the training of the model, we compared the selection of some parameters and fine tuned the models. For the four architectures described above, we evaluated several design choices including learning rate, epoch, and dropout probability.

#### Epoch and Learning Rate

The epoch is the number of iterations during training. The learning rate determines the convergence speed and result accuracy of the model. Taking the CNN and LSTM as examples, the training results of different epochs and learning rates were adjusted and shown in Table 5.

**Table 5:** The influence of different epoch and learning rates for the DNN and CNN models.

**Epoch**	**Learning rate**	**DNN**	**CNN**
3	0.001	55.3	26.3
3	0.05	59.2	35.9
3	0.01	62.5	43.2
10	0.001	63.3	58.3
10	0.05	63.4	62.5
10	0.01	63.4	75.4
20	0.001	63.3	80.3
20	0.05	63.4	78.5
20	0.01	63.4	79.2

It can be seen that the DNN model converged to optimal with fewer epochs and a higher learning rate. In contrast, the CNN model needed more epochs to learn. We chose 10 epoches and 0.01 as the learning rate for the DNN model. For the CNN model, we set 20 epoches and used the self-regulated learning rate callback function to autonomously adjust according to the training condition.

#### Dropout Probability

Since the dropout setting is very important for the gradient descent process of the LSTM model, we set the dropout probability at 20, 50, and 80%, respectively, and tested the LSTM model and CNN-LSTM model.

It can be seen in Table 6 that reducing the parameters by ~50% each time was appropriate.

**Table 6:** The influence of dropout probability for the LSTM and CNN-LSTM models.

**Dropout probability (%)**	**LSTM**	**CNN-LSTM**
20	0.58	0.62
50	0.71	0.89
90	0.51	0.48

### Result Discussion

The experimental results show that the CNN model and CNN-LSTM had better performance in emotion recognition classification, and were consistently more stable and had higher accuracy in RAW data than STD data. This result verified the advantages of the end-to-end deep learning mode we mentioned earlier, and proved that the CNN can be regarded as a feature extractor in end-to-end classification, which can automatically extract hidden features in EEG signals. It comes to a conclusion that the CNN feature extracter is more suitable for emotion recognition than manual feature extraction in emotion recognition based on EEG signals of the DEAP dataset.

The performance of the DNN model was not as good as the other complex models, but the training speed was fast. The DNN model could achieve optimal performance in fewer epochs and at a faster learning rate. The LSTM model was not as stable as the CNN and CNN-LSTM models. Moreover, with the same number of parameters, the training speed of the LSTM was much slower and it struggled to achieve convergence.

In addition, we found that the DNN model only needed a few training epochs to achieve convergence. The method of automatically adjusting the learning rate was suitable for the CNN and CNN-LSTM models. It was better to set the dropout rate of the LSTM at a medium level.

## Conclusion

In this paper, several deep learning models for the classification of emotions were established and their performance was verified on the DEAP dataset. It was concluded that the CNN model or CNN-LSTM hybrid models were more effective in emotional classification than traditional machine learning methods. In particular, the automatical feature extraction of EEG signals was proven to have high performance in end-to-end multi-dimensional emotion recognition.

In the next step of research, we will try to obtain more data on EEG signals and implement other EEG-based emotional recognition models with more variables considered.

## Data Availability Statement

Publicly available datasets were analyzed in this study. This data can be found at: DEAPdataset http://www.eecs.qmul.ac.uk/mmv/datasets/deap/index.html.

## Author Contributions

YZ was responsible for writing the manuscript and carrying out experiments. JC, YuxC, YunC, and DL collected data for the experiments. LY checked the English grammer of the article. All authors contributed to the article and approved the submitted version.

## Conflict of Interest

The authors declare that the research was conducted in the absence of any commercial or financial relationships that could be construed as a potential conflict of interest.
